# P-2302. Efficacy of Universal vs Targeted Antifungal Prophylaxis for the Prevention of Invasive Fungal Infections after Lung Transplant

**DOI:** 10.1093/ofid/ofae631.2455

**Published:** 2025-01-29

**Authors:** Ashmead-Meers Claire, Marisa H Miceli, Kathleen A Linder, Carol A Kauffman, Blair Richards

**Affiliations:** University of Michigan, Ann Arbor, Michigan; University of Michigan, Ann Arbor, Michigan; Ann Arbor VAMC, Ann Arbor, Michigan; University of Michigan, Ann Arbor, Michigan; University of Michigan Medical School, Ann Arbor, Michigan

## Abstract

**Background:**

IFI are associated with high morbidity and mortality among LTx patients (pt). Preventive strategies are commonly used, but the optimal AP approach is unclear. Over the last 10 years, our LTx program has transitioned from universal AP with itraconazole (ITR), to targeted AP, and now universal AP with posaconazole (POS). We compared the efficacy of these strategies.

**Methods:**

All adult LTx pt at U Michigan from 7/1/2014-2/28/2023 were followed for 12 months (mo) post-LTx. Pt were divided into 3 cohorts based on AP regimen: cohort 1 - universal ITR for 6 mo; cohort 2 - targeted voriconazole (VOR) for 3 mo if colonized with *Aspergillus* or fluconazole (FLU) for 14 days (d) if colonized with *Candida*; cohort 3 - universal POS for 6 mo. Demographics, LTx characteristics, IFI defined by MSGERC criteria, breakthrough IFI (B-IFI) defined by MSGERC/ECMM criteria, and mortality were noted.

**Results:**

Of 134 pt, 86 (64%) were men, and 101 (75%) had double-LTx. Pulmonary fibrosis was the most common reason for LTx (47,35%). There were 59 (44%), 29 (22%), and 46 (34%) pt in the universal ITR, universal POS, and targeted cohorts, respectively. A total of 23 proven/probable IFI occurred: 14 invasive pulmonary aspergillosis (IPA), 7 invasive candidiasis (IC), 1 cryptococcal pneumonia (CP) and 1 hyaline mold IFI. IFI occurred at a median of 98 d (range 1–322 d). IFI occurred in 6/59 (10%) receiving universal ITR (all but one of whom had IPA, all B-IFI); 5/29 (17%) receiving universal POS (3 IC and 2 IPA, all B-IFI); and 12/46 (26%) in the targeted AP cohort (7 IPA, 4 IC, 1 CP). Of the 12 pt with IFI in the targeted cohort, 10 did not meet criteria for use of AP and thus were not given VOR or FLU; 7/10 developed IPA and 2/10 IC. No IPA occurred in 5 pt who received VOR AP, but 2 pt on FLU AP developed breakthrough IC. Kaplan-Meier curve showed a trend toward IFI-free survival at 12 mo to be greater among pt receiving universal ITR (0.89) and universal POS (0.83) vs. targeted AP (0.73), p=0.16. All-cause mortality was 10% (14/134) and did not differ among the 3 AP cohorts, p=.49. Only 1 death (IC) was caused by IFI.

**Conclusion:**

Universal AP using POS or ITR may be a more effective strategy than targeted AP for prevention of IFI post-LTx, but this comes with an increased risk for harder-to-treat B-IFI.

**Disclosures:**

Marisa H. Miceli, MD, AN2: PI in clinical trial|F2G: PI in clinical trial|Pulmocide: PI in clinical trial|Scynexis: Advisor/Consultant|Scynexis: PI in clinical trial

